# An Uncommon Case of Solitary Peripheral Osteoma in the Mandible

**DOI:** 10.1155/2015/319738

**Published:** 2015-12-14

**Authors:** Rohit Agrawal, Shipra Agrawal, Shitij Bhargava, Mahesh Motlani, Rahul Agrawal

**Affiliations:** ^1^Department of Oral Medicine and Radiology, Maharana Pratap College of Dentistry and Research Centre, Gwalior 475001, India; ^2^Department of Conservative Dentistry and Endodontics, Maharana Pratap College of Dentistry and Research Centre, Gwalior 475001, India; ^3^Private practice, Bhargava Industries, Industrial State, Govinpura Bhopal 462023, India; ^4^Department of Conservative Dentistry and Endodontics, Chhattisgarh Dental College and Research Institute, Rajnandgaon 491441, India; ^5^Department of ENT, Agrawal Hospital and Research Institute, H-204, Madhav Nagar, Gwalior 474001, India

## Abstract

Osteoma is a benign osteogenic lesion which is composed of well differentiated mature compact and/or cancellous bone that proliferates continuously. Its prevalence is 4%. Its pathogenesis is still controversial. Solitary peripheral osteoma of craniofacial region is a rare finding. We report a case of 30-year-old female having solitary peripheral osteoma present on the lingual cortex of the left posterior mandible which was initially asymptomatic but now is causing discomfort while chewing and not associated with Gardner's syndrome. We also laid emphasis on its clinical, differential diagnosis, radiological, surgical, and histopathological features. The aim of this paper is to present an uncommon case of solitary peripheral osteoma in the mandible along with analysis of literature for peripheral osteomas of jaws and to contribute to the knowledge concerning the pathogenesis, differential diagnosis, and management of these lesions.

## 1. Introduction

Osteomas are uncommon, slow-growing, benign osteogenic neoplasms that arise most frequently in the craniofacial skeleton [1, 2]. Osteomas of jaws may arise on the surface of the bone (peripheral, periosteal, exophytic, or parosteal) or they may be located in the medullary bone (endosteal or central). Peripheral type arises from the periosteum while central osteoma arises from the endosteum. Rarely soft tissue osteoma can also be seen within a muscle [3, 4]. The pathogenesis of the peripheral osteoma is unclear. It has been considered to be a true neoplasm, developmental anomaly, or reactive lesion triggered by trauma, muscle traction, or infection [3–6]. Endocrine factors have been considered to be one of the etiological factors for osteoma [7]. They are categorized as central, peripheral, or extraskeletal according to location [8–10]. Osteoma is the most common benign tumor of the nose and paranasal sinuses and the most common neoplasm of the frontal sinus [11–13]. Solitary peripheral osteomas affecting the mandible are rare [6, 8, 14], mostly occurring in young adults [15], which affect equally men and women [16]. There is no predilection for age or sex [17] and it may develop from 4.8 months to 50 years of age [18, 19]. Generally it is asymptomatic [3, 4, 15]. They appear radiographically as well-circumscribed radiopaque masses that appear round or ovoid in shape [4, 14, 15, 20]. Histologically, they can be compact osteoma, cancellous osteoma, and mixed osteoma [15]. Patients with multiple osteomas affecting the mandible should be evaluated for Gardner's syndrome [18]. The triad of colorectal polyposis, skeletal abnormalities, that is, multiple osteomas, and multiple impacted or supernumerary teeth are commonly present in patients suffering from this syndrome [15]. Osteomas are managed by surgical excision but it is indicated only in symptomatic patients [21]. The solitary peripheral osteoma of the mandible is a rare finding, with only 26 cases found till now not related to Gardner's syndrome [14].

Aim of this paper is to present an uncommon case of solitary peripheral osteoma in the mandible along with analysis of literature for peripheral osteomas of jaws and to contribute to the knowledge concerning the pathogenesis, differential diagnosis, and management of these lesions.

## 2. Case Report

A 30-year-old female had reported to our department with a chief complaint of a swelling on the inner side of lower left posterior region of the mandible since 8 years. Her history revealed that 8 years back she developed a small swelling measuring around 5 mm in diameter which slowly progressed to current size. The swelling was asymptomatic, but, as the swelling had increased in its size, now the patient experiences discomfort while chewing food and wants to have it removed. The patient did not have any significant medical and family history and also gave no history of trauma. Physical examination did not reveal any kind of similar swelling in any other region of the whole body and also patient did not have any significant family and medical history of such swellings so Gardner's syndrome was ruled out.

**Figure 1:**
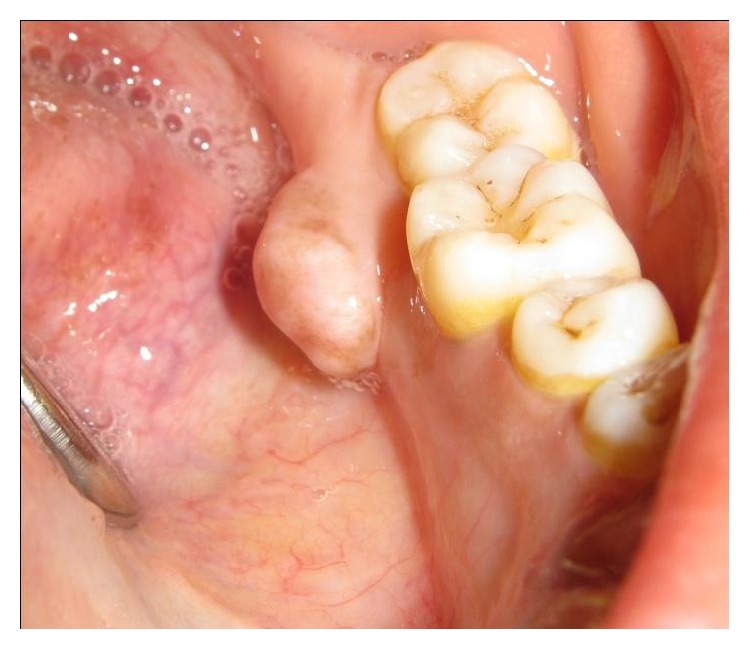
Intraoral picture of the lesion.

Intraorally a solitary oval shaped well defined pedunculated swelling was present on the lingual aspect of teeth 36 and 37 which measured 1 cm in diameter (Figure 1). The overlying and surrounding mucosa was normal. On palpation it was lobulated, nontender, and bony hard in consistency.

**Figure 2:**
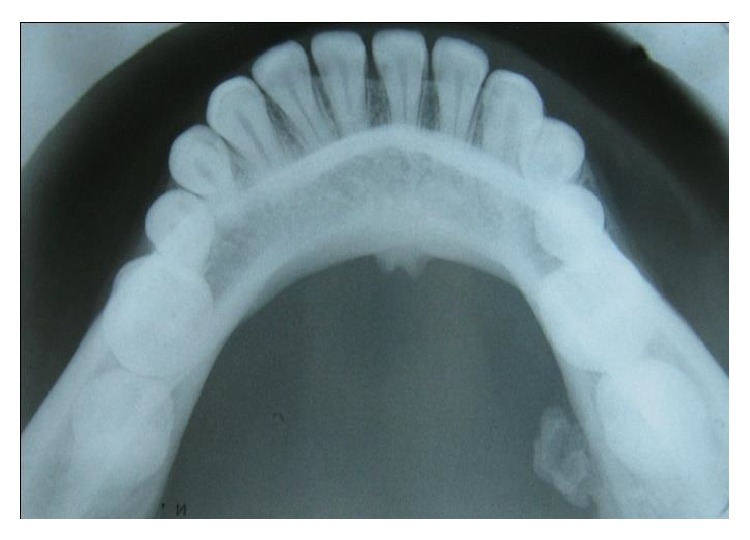
Mandibular occlusal cross-sectional radiograph.

Mandibular occlusal cross-sectional radiograph showed a well defined irregular shaped radiopacity attached to the lingual cortex of 37 with a stalk (Figure 2). Its radiodensity was equivalent to that of the adjacent body of the mandible. Differential diagnoses of exostosis, peripheral ossifying fibroma, osteoid osteoma, osteoblastoma, and osteosarcoma were included. Patient consent was taken after careful explanation of the surgical procedure used and the risks and benefits. Excisional biopsy was performed under local anesthesia. Intraoral incision was performed with periosteal elevation. It was removed completely with the help of rotary instrument and chisel.

**Figure 3:**
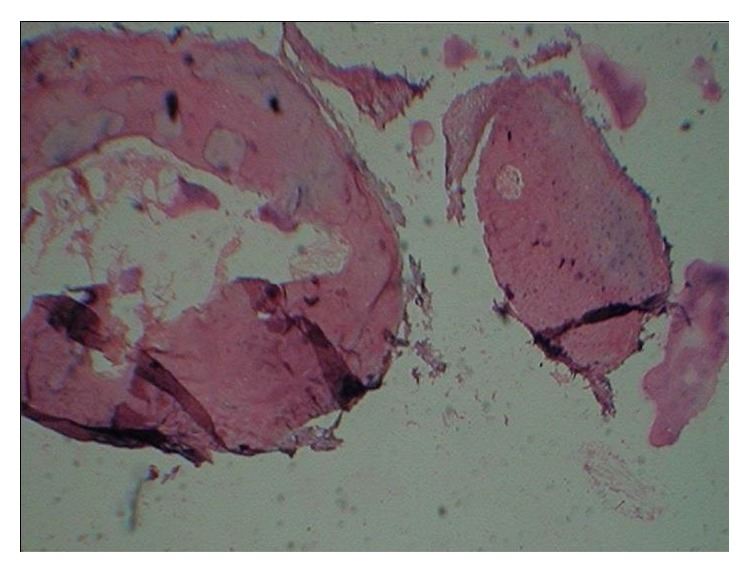
10x scanner view H&E stain.

The histopathological evaluation of the excised specimen showed that the lesional tissue was composed of dense compact bone tissue exhibiting concentric lamellae and osteocytes within osteocyte lacunae (Figure 3). These microscopic findings along with clinical and radiological features guided us to arrive at the final diagnosis of Compact Type Osteoma.

## 3. Discussion

Osteoma is defined by World Health Organization (WHO) as a benign lesion consisting of well differentiated mature bone tissue with a predominantly laminar structure and showing very slow growth [22]. It is not clear whether osteomas are benign neoplasms or hamartomas [23]. But WHO, in its latest classification of diseases, reports osteoma as a benign osseous neoplasm, clearly distinct from exostoses and tori, which are hamartomas, an approach adopted by many authors. Their etiology is obscure [8, 14, 24]. Fetissof, as reported by Varboncoeur et al. [10], considered these tumors to arise either from embryological cartilaginous rests or from persistent embryological periosteum cells. According to the developmental or embryological theory, osteomas would then originate from the suture between bones with different embryological derivation (membranous/enchondral) [10]. Other possible etiological factors are inflammation [25], trauma, and endocrinal pathologies [7]. Kaplan et al. [6] suggested that a combination of trauma and muscle traction may play a role in its development. But none of these hypotheses suited the etiology of osteoma as seen in our case. Regezi and Sciubba [26] said that none of the proposed hypotheses related to the etiology of these lesions has been proven.

Histologically they can be divided into three types: compact or ivory or eburnated osteoma (consisting of dense, compact bone with few marrow spaces and difficult to resect), cancellous or trabecular or mature osteoma or osteoma spongiosum (consisting of soft, spongy bone and fibrofatty marrow), and mixed-type osteomas [6, 14, 17, 18].

Solitary peripheral osteomas (PO) of the jaws are rare, commonly occurring in young adults [23] with no gender predilection [6, 17, 22, 25]. Our patient was 30 years old. Osteomas are usually asymptomatic and often remain undetected unless incidentally found on a routine radiographic investigation or until they cause facial asymmetry or functional impairment leading to malocclusion or when it becomes symptomatic [17, 20]. The most common gnathic locations are the body of mandible posterior to premolar on the lingual surface and the condyles. Other less common locations are angle, coronoid process, ramus, external auditory canal, orbit, temporal bone, maxilla, zygomatic arch, and pterygoid processes [14, 25].

Multiple osteomas of the jaws along with other pathologies like colorectal polyps with high malignity, cutaneous fibromas, congenital retinal pigment hypertrophies, and multiple impacted or supernumerary teeth, enostoses, or epidermal cysts, are characteristic findings in Gardner's syndrome (familial adenomatous polyposis) which is an autosomal dominant disease caused by a mutation in the* APC* (adenomatous polyposis coli) tumor suppressor gene [8, 15, 27]. Osteomas rarely can also be seen in Haberland syndrome (Encephalocraniocutaneous Lipomatosis) [28].

Radiologically PO appear as well defined radiopaque mushroom or oval shaped masses with a narrow contact area near vestibule with density similar to normal bone [14]. They can either be pedunculated as seen in our case or sessile [25]. Traditional radiographic imaging like occlusal radiograph is generally sufficient to diagnose an osteoma like in our case. But if they are present in other deeper areas of the craniofacial region, then we have to go for higher imaging modalities like panoramic radiographs, Waters view, computerized tomography (CT), magnetic resonance imaging (MRI), or cone beam computed tomography (CBCT) [14, 16, 18, 19, 25]. Spiral CT gives the three-dimensional reconstruction of osteoma. With the aid of Radionuclide bone imaging one can differentiate between an actively growing lesion (“hot”) and a stable lesion (“cold”). Cancellous osteoma is vascular while the compact osteoma is avascular which can be seen by intravenous angiography [29].

The differential diagnosis of osteomas includes exostoses, osteochondroma, osteoid osteoma, periosteal osteoblastoma, parosteal osteosarcoma, peripheral ossifying fibroma, Paget's disease, fibrous dysplasia, and odontoma [17, 20]. Exostoses are hamartomas, commonly located in the mandible and the palate. These tend to stop growing after puberty while osteomas continue to grow slowly over time and appear as well circumscribed, lobulated, radiopaque masses [4]. Osteochondromas are composed of areas of endochondral ossification, calcified cartilage, and fatty or haematopoietic marrow in the trabecular spaces and merge well with the cortex of the host bone [17]. Osteoid osteoma has a rapid growth, is frequently painful, and contains highly vascular cellular tissue with osteoid tissue [9, 17]. Periosteal osteoblastoma also presents as a rapidly growing painful round or ovoid heterogeneous mass attached to the cortex [17].

Parosteal osteosarcoma is commonly seen in posterior mandible, homogeneous or heterogeneous, ill defined lobulated sclerotic mass. At periphery it can have either sun burst appearance or formation of Codman's triangle [30].

Peripheral ossifying fibroma is found exclusively on gingiva and is a reactive lesion commonly occurring in the maxillary anterior region which is firm in consistency [9]. Paget's disease is more common in femur, skull, and vertebrae and less common in jaws and it occurs in older age group and is almost always bilateral when it involves jaws; maxilla is commonly involved. The affected bone is deformed and enlarged [31]. Fibrous dysplasia commonly involves the posterior maxilla and radiographically it appears as diffused radiopaque mass which can have varied pattern like ground glass, orange peel, or cotton wool [32]. Odontomas often interfere with eruption of permanent teeth and have radiopacity equivalent to that of tooth and are always surrounded by a radiolucent capsule [33].

## 4. Conclusion

Patient with osteoma should be evaluated properly clinically and their family and medical history should be recorded thoroughly to rule out Gardner's syndrome [18]. Asymptomatic osteomas should be managed conservatively while surgery is indicated in cases associated with other functional pathologies but periodic clinical and radiographic follow-up after surgical excision of a PO is a must.
